# Supplementary material for: Use of Health Services and Rehabilitation before and after the Beginning of Long-Term Sickness Absence—Comparing the Use by Employment and Disability Pension Transition after the Sickness Absence in Finland
Source: Int J Environ Res Public Health. 2022 Apr 20;19(9):4990. doi: 10.3390/ijerph19094990 (PMC9099499; doi:10.3390/ijerph19094990)
Supplement: Supplementary file 1 [file ijerph-19-04990-s001.zip › ijerph-1653435-supplementary.pdf]

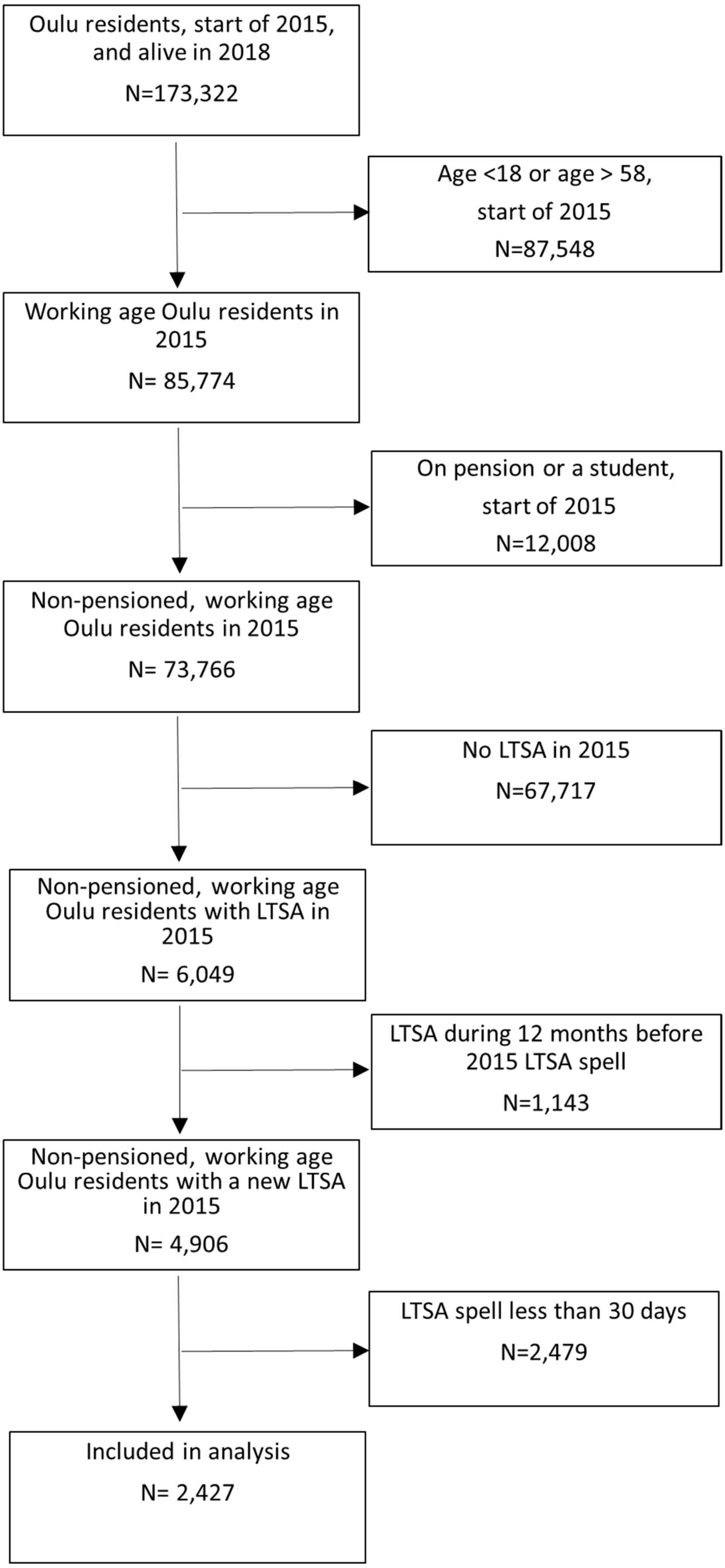

Figure S1. Flowchart of subjects who met inclusion criteria.

Table S1. The LTSA groups.

	Label	Definition	N
Group 1	Transfer to DP after LTSA	Transitioned to disability pension by the end of the follow-up	198
Group 2	Mostly employed after LTSA	Employed at least half of the last 12 follow-up months (third follow-up year)	1,639
Group 3	Some employment after LTSA	Employed at least 30 calendar days, but less than half of the last 12 months (third follow-up year)	169
Group 4	Not employed after LTSA	Employed less than 30 days during the last 12 follow-up months (third follow-up year)	430

Table S2. The LTSA groups and covariates associated with the expected number of outpatient health care visits after the start of LTSA in negative binomial regression analysis models (Incident rate ratios [IRR], predicted means and 95 % confidence intervals [CI]). Group 1 as the reference group.

	M1				M2				M3			
	**IRR**	**95% CI**	**Predicted means**	**95% CI**	**IRR**	**95% CI**	**Predicted means**	**95% CI**	**IRR**	**95% CI**	**Predicted means**	**95% CI**
LTSA group												
G1 - Transfer to DP after LTSA	1.00		7.56	6.75–8.36	1.00		7.43	6.62–8.23	1.00		4.87	4.36–5.38
G2 - Mostly employed after LTSA	0.48	0.43–0.54	3.65	3.51–3.79	0.49	0.44–0.55	3.66	3.51–3.81	0.78	0.69–0.87	3.79	3.65–3.94
G3 - Some employment after LTSA	0.52	0.45–0.62	3.96	3.49–4.43	0.53	0.45–0.62	3.94	3.47–4.40	0.76	0.65–0.88	3.69	3.28–4.10
G4 - Not employed after LTSA	0.74	0.65–0.84	5.57	5.18–5.97	0.74	0.65–0.85	5.51	5.06–5.96	0.87	0.77–0.98	4.22	3.89–4.55
Sex												
Male	1.00		3.61	3.43–3.78	1.00		3.65	3.47–3.83	1.00		3.54	3.38–3.71
Female	1.30	1.22–1.38	4.68	4.49–4.86	1.26	1.18–1.35	4.61	4.42–4.80	1.20	1.13–1.27	4.25	4.09–4.41
Age group												
18–30	1.00		3.94	3.66–4.23	1.00		3.83	3.54–4.13	1.00		3.79	3.51–4.06
31-40	1.06	0.96–1.16	4.17	3.90–4.43	1.08	0.97–.1.19	4.13	3.86–4.39	1.04	0.95–1.14	3.92	3.69–4.15
41-50	1.14	1.04–1.26	4.52	4.25–4.78	1.18	1.07–1.31	4.54	4.27–4.81	1.08	0.99–1.19	4.10	3.88–4.33

51-58	1.04	0.94–1.14	4.09	3.84–4.34	1.08	0.97–1.20	4.14	3.88–4.40	1.03	0.93–1.14	3.90	3.67–4.12
Marital status												
Married					1.00		4.09	3.90–4.28	1.00		3.89	3.73–4.06
Unmarried					1.04	0.97–1.13	4.27	4.03–4.50	1.03	0.96–1.10	4.00	3.80–4.21
Divorced / separated / widowed					1.03	0.95–1.13	4.22	3.90–4.54	1.00	0.92–1.08	3.90	3.63–4.17
Occupational class												
Upper non-manual employee					1.00		3.97	3.64–4.29	1.00		3.77	3.48–4.05
Lower non-manual employee					1.12	1.02–1.23	4.44	4.19–4.70	1.14	1.04–1.25	4.29	4.07–4.52
Manual worker					1.01	0.91–1.12	4.00	3.73–4.27	1.01	0.91–1.11	3.80	3.56–4.03
Entrepreneur					0.80	0.68–0.94	3.16	2.71–3.62	0.73	0.62–.85	2.74	2.37–3.11
Other					1.11	0.97–1.26	4.40	4.01–4.78	1.07	0.95–1.20	4.01	3.69–4.34
Labour market status at the start of LTSA												
Employed					1.00		4.19	4.03–4.36	1.00		4.05	3.91–4.20
Unemployed					0.97	0.87–1.09	4.09	3.69–4.49	0.88	0.79–0.98	3.56	3.23–3.88
Other					1.00	0.86–1.17	4.21	3.63–4.80	0.88	0.77–1.01	3.57	3.10–4.02
Chronic or severe diseases												
No									1.00		3.60	3.47–3.72
One disease									1.32	1.23–1.42	4.76	4.47–5.06
Multiple diseases									1.54	1.40–1.70	5.55	5.04–6.05
LTSA length												
Under 2 months									1.00		2.68	2.53–2.83
Two months to 11 months									1.53	1.42–1.64	4.09	3.93–4.26
Maximum length									2.40	2.18–2.63	6.43	6.01–6.85
LTSA diagnosis group												
Mental LTSA									1.00		4.93	4.62–5.23
Musculoskeletal LTSA									0.75	0.69–.81	3.68	3.47–3.89
Other diagnosis LTSA									0.76	0.70–.81	3.73	3.58–3.87

M1: Adjusted for sex, age group,

M2: Adjusted for sex, age group, marital status, occupational class, labour market status at the start of LTSA,

M3: Adjusted for sex, age group, marital status, occupational class, labour market status at the start of LTSA, chronic diseases, LTSA length, LTSA diagnosis group (fully adjusted model).

Table S3. The LTSA groups and covariates associated with the expected number of days in inpatient care after the start of LTSA in negative binomial regression analysis models (Incident rate ratios [IRR], predicted means and 95 % confidence intervals [CI]). Group 1 as the reference group.

	M1				M2				M3			
	**IRR**	**95% CI**	**Predicted means**	**95% CI**	**IRR**	**95% CI**	**Predicted means**	**95% CI**	**IRR**	**95% CI**	**Predicted means**	**95% CI**
LTSA group									IRR	CI 95%		
G1 - Transfer to DP after LTSA	1.00		1.95	1.47–2.43	1.00		1.79	1.34–2.23	1.00		0.84	0.62–1.06
G2 - Mostly employed after LTSA	0.16	0.12–0.21	0.31	0.28–0.35	0.18	0.14–0.24	0.32	0.28–0.36	0.35	0.26–0.47	0.29	0.26–0.33
G3 - Some employment after LTSA	0.27	0.18–0.40	0.52	0.36–0.68	0.27	0.18–0.40	0.49	0.34–0.63	0.47	0.32–0.71	0.40	0.28–0.52
G4 - Not employed after LTSA	0.39	0.29–0.54	0.77	0.63–0.90	0.35	0.25–0.50	0.62	0.50–0.75	0.50	0.37–0.69	0.42	0.33–0.51
Sex												
Male	1.00		0.44	0.38–0.50	1.00		0.43	0.37–0.49	1.00		0.36	0.31–0.41
Female	1.00	0.84–1.18	0.44	0.39–0.49	0.97	0.81–1.17	0.42	0.37–0.47	0.95	0.79–1.13	0.34	0.30–0.38
Age group												
18–30	1.00		0.52	0.42–0.61	1.00		0.55	0.44–0.66	1.00		0.50	0.40–0.60
31-40	0.92	0.72–1.18	0.48	0.39–0.56	0.82	0.63–1.06	0.45	0.38–0.53	0.73	0.56–0.95	0.36	0.30–0.43
41-50	0.82	0.64–1.05	0.43	0.36–0.50	0.73	0.56–0.97	0.41	0.34–0.47	0.65	0.49–0.86	0.32	0.27–0.38
51-58	0.73	0.56–0.94	0.38	0.31–0.44	0.63	0.50–0.83	0.35	0.28–0.41	0.56	0.41–0.74	0.28	0.23–0.33
Marital status												
Married					1.00		0.45	0.39–0.51	1.00		0.37	0.32–0.42
Unmarried					0.83	0.67–1.02	0.37	0.32–0.43	0.85	0.69–1.04	0.32	0.27–0.37
Divorced / separated / widowed					1.04	0.82–1.31	0.47	0.37–0.56	0.95	0.75–1.20	0.36	0.28–0.43
Occupational class												
Upper non-manual employee					1.00		0.56	0.44–0.67	1.00		0.44	0.34–0.53

Lower non-manual employee	0.70	0.54−0.90	0.39	0.32−0.45	0.75	0.58−0.98	0.33	0.28−0.38
Manual worker	0.54	0.41−0.73	0.30	0.24−0.36	0.57	0.42−0.76	0.25	0.20−0.30
Entrepreneur	0.76	0.50−1.16	0.42	0.27−0.58	0.66	0.43−1.02	0.29	0.18−0.40
Other	1.03	0.73−1.44	0.57	0.45−0.70	1.10	0.79−1.53	0.48	0.37−0.59
Labour market status at the start of LTSA								
Employed	1.00		0.41	0.37−0.46	1.00		0.35	0.31−0.39
Unemployed	1.14	0.86−1.52	0.47	0.36−0.59	0.93	0.70−1.25	0.33	0.25−0.41
Other	1.04	0.72−1.50	0.43	0.28−0.58	0.91	0.64−1.31	0.32	0.21−0.43
Chronic or severe diseases								
No					1.00		0.30	0.26−0.33
One disease					1.86	1.53−2.26	0.55	0.46−0.65
Multiple diseases					1.60	1.21−2.12	0.48	0.35−0.60
LTSA length								
Under 2 months					1.00		0.17	0.13−0.20
Two months to 11 months					2.42	1.92−3.06	0.41	0.36−0.45
Maximum length					4.39	3.32−5.80	0.73	0.60−0.87
LTSA diagnosis group								
Mental LTSA					1.00		0.38	0.31−0.45
Musculoskeletal LTSA					0.53	0.40−0.69	0.20	0.16−0.24
Other diagnosis LTSA					1.16	0.94−1.44	0.44	0.39−0.49

M1: Adjusted for sex, age group,

M2: Adjusted for sex, age group, marital status, occupational class, labour market status at the start of LTSA

M3: Adjusted for sex, age group, marital status, occupational class, labour market status at the start of LTSA, chronic diseases, LTSA length, LTSA diagnosis group (fully adjusted model).

Table S4. The LTSA groups and covariates associated with the expected number of days in rehabilitation after the start of LTSA in negative binomial regression analysis models (Incident rate ratios [IRR], predicted means and 95 % confidence intervals [CI]). Group 1 as the reference group.

	M1				M2				M3			
	**IRR**	**95% CI**	**Predicted means**	**95% CI**	**IRR**	**95% CI**	**Predicte d means**	**95% CI**	**IRR**	**95% CI**	**Predicte d means**	**95% CI**
LTSA group									IRR	CI 95%		
G1 - Transfer to DP after LTSA	1.00		1.59	0.46–2.73	1.00		1.08	0.30–1.87	1.00		0.40	0.12–0.68
G2 - Mostly employed after LTSA	0.70	0.33–1.47	1.11	0.86–1.36	0.98	0.46–2.09	1.06	0.82–1.30	1.91	0.91–4.03	0.76	0.60–0.93
G3 - Some employment after LTSA	1.70	0.61–4.73	2.72	0.82–4.63	2.23	0.78–6.34	2.41	0.65–4.17	3.23	1.14–9.10	1.29	0.37–2.21
G4 - Not employed after LTSA	2.77	1.17–6.56	4.42	2.42–6.40	3.77	1.55–9.16	4.08	2.21–5.59	5.56	2.36–13.12	2.22	1.28–3.16
Sex												
Male	1.00		1.47	1.05–1.90	1.00		1.23	0.87–1.59	1.00		0.79	0.56–1.02
Female	1.10	0.76–1.61	1.63	1.23–2.03	1.31	0.87–1.95	1.60	1.20–2.00	1.28	0.86–1.90	1.01	0.77–1.25
Age group												
18–30	1.00		1.72	0.98–2.45	1.00		1.58	0.79–2.36	1.00		1.65	0.87–2.43
31-40	1.16	0.65–2.05	1.99	1.23–2.75	.1.10	0.58–2.10	1.74	1.05–2.43	0.50	0.26–0.93	0.82	0.50–1.14
41-50	0.93	0.53–1.62	1.59	1.02–2.16	0.91	0.48–1.75	1.44	0.91–1.97	0.47	0.25–0.87	0.77	0.49–1.05
51-58	0.68	0.38–1.22	1.17	0.74–1.60	0.72	0.36–1.42	1.13	0.70–1.56	0.47	0.24–0.89	0.77	0.49–1.05
Marital status												
Married					1.00		1.60	1.13–2.06	1.00		1.21	0.88–1.54
Unmarried					0.74	0.45–1.19	1.18	0.77–1.58	0.47	0.30–0.75	0.57	0.38–.76
Divorced / separated / widowed					1.01	0.58–1.75	1.61	0.85–2.38	0.97	0.56–1.69	1.17	0.62–1.72
Occupational class												
Upper non-manual employee					1.00		0.72	0.35–1.09	1.00		0.63	0.33–0.94
Lower non-manual employee					2.25	1.24–4.09	1.62	1.09–2.16	1.93	1.08–3.45	1.23	0.83–1.62
Manual worker					3.32	1.73–6.40	2.39	1.45–3.34	2.43	1.27–4.65	1.54	0.95–2.14
Entrepreneur					2.14	0.83–5.54	1.54	0.26–2.83	1.15	0.45–2.94	0.73	0.14–1.32
Other					1.52	0.74–3.16	1.10	0.59–1.62	0.71	0.35–1.43	0.45	0.24–0.66
Labour market status at the start of LTSA												
Employed					1.00		1.33	1.04–1.63	1.00		0.77	0.60–0.93
Unemployed					1.18	0.65–2.14	1.57	0.76–2.39	1.74	0.94–3.22	1.34	0.63–2.04
Other					2.18	0.92–5.20	2.91	0.51–5.31	3.86	1-62–9.16	2.96	0.55–5.38

Chronic or severe diseases				
No	1.00		0.80	0.63−.97
One disease	1.10	0.68−1.79	0.88	0.51−1.25
Multiple diseases	3.81	1.90−7.62	3.05	1.08−5.01
LTSA length				
Under 2 months	1.00		0.24	0.15−0.33
Two months to 11 months	4.76	2.88−7.88	1.15	0.84−1.46
Maximum length	16.31	9.38−28.37	3.94	2.41−5.48
LTSA diagnosis group				
Mental LTSA	1.00		1.14	0.62−1.65
Musculoskeletal LTSA	1.39	0.76−2.53	1.58	1.02−2.14
Other diagnosis LTSA	0.56	0.32−0.97	0.63	0.47−0.80

M1: Adjusted for sex, age group,

M2: Adjusted for sex, age group, marital status, occupational class, labour market status at the start of LTSA

M3: Adjusted for sex, age group, marital status, occupational class, labour market status at the start of LTSA, chronic diseases, LTSA length, LTSA diagnosis group (fully adjusted model).
